# Anti-neuroinflammatory therapy for non-pulsatile tinnitus in patients with sinus vascular anomalies: preliminary result on two cases

**DOI:** 10.3389/fneur.2025.1558196

**Published:** 2025-07-23

**Authors:** Arianna Di Stadio, Daniela Messineo, Iole Indovina, Giovanni Motta, Massimo Ralli, Michael J. Brenner

**Affiliations:** ^1^Department of Otolaryngology, University of Campania “Luigi Vanvitelli”, Naples, Italy; ^2^Dipartimento di Scienze Radiologiche, Oncologiche ed Anatomo Patologiche, University La Sapienza, Rome, Italy; ^3^Department of Systems Medicine and Centre for Space BioMedicine, University of Rome Tor Vergata, Rome, Italy; ^4^International Medical University, Unicamillus, Rome, Italy; ^5^Department of Otolaryngology, Head and Neck Surgery, University of Michigan Medical School, Ann Arbor, MI, United States

**Keywords:** tinnitus, neuroinflammation, sigmoid sinus, hyperactive brain, palmythoylethanolamide, Luteolin

## Abstract

**Background:**

Vascular anomalies are commonly associated with pulsatile tinnitus, but their potential role in triggering non-pulsatile tinnitus through neuroinflammation is not well established. Furthermore, few studies have investigated non-pulsatile tinnitus associated with altered cerebrovascular supply and whether anti-neuroinflammatory therapy might alleviate symptoms.

**Materials and methods:**

We describe two patients presenting with non-pulsatile tinnitus and sigmoid sinus stenosis confirmed by imaging, with other causes systematically excluded.

**Results:**

Magnetic resonance venography demonstrated severe stenosis of the transverse and sigmoid sinuses, alongside compensatory ipsilateral vascular dilatation. These structural abnormalities suggested disrupted venous blood flow, contributing to the neuroinflammatory processes associated with non-pulsatile tinnitus. Following treatment with an anti-inflammatory molecule, both patients reported a substantial reduction in tinnitus severity.

**Conclusion:**

This study supports the potential of anti-inflammatory treatments in managing non-pulsatile tinnitus linked to sinus vascular anomalies. Further research is warranted to elucidate these relationships and confirm therapeutic efficacy of anti-inflammatory agents.

## Introduction

Tinnitus affects 8 to 25.3% of the US population (1), with similar prevalences in European countries, ranging from 8.7% in Ireland to 28.3% in Bulgaria (2). A recent systematic review found a 14.4% pooled prevalence of any type of tinnitus among adults, with a range between 4.1 and 37.2% (3). Overall, tinnitus affects more than 740 million adults globally and is perceived as a major problem for more than 120 million people, mostly aged 65 years or older (3).

Tinnitus has a multi-factorial origin (4). In most cases, it is initiated by injury to the peripheral auditory system (5), primarily attributable to cochlear damage within the organ of Corti. However, tinnitus can also be an early sign of neuro-inflammatory disorders like multiple sclerosis (6). Central tinnitus arises from hyperactivation of auditory cortex (4), and this hyperactivation can occur gradually or abruptly as with sudden hearing loss (6, 7); it generally causes central re-organization, as evidenced by EEG studies (8).

Neuroinflammation appears capable of inducing auditory hyperactivation characteristic of central tinnitus (5). In animal studies, noise-induced parvalbumin-positive inhibitory neuron loss in the auditory cortex results in an excitation–inhibition imbalance in the central auditory pathway (9). The neuroinflammation causes both acute and chronic tinnitus (10); thus, some authors have proposed that reducing neuroinflammation might alleviate tinnitus (4).

Alteration of cerebrovascular supply can induce brain neuroinflammation, as shown by studies of patients with cardiovascular disease and atherosclerosis (10). Neuroradiological studies demonstrate a correlation between hypoplasia or stenosis of sigmoid sinus and tinnitus (11); moreover, this anatomic finding has been associated with brain hypoperfusion and changes in white matter (12). Venous stenosis or hypoplasia leads to impaired blood flow that is analogous to a traffic jam within the cerebral circulation. When venous outflow is restricted, venous blood accumulates proximal to the site of narrowing, while arterial inflow continues largely unabated. This imbalance results in increased venous pressure—referred to as venous hypertension—in the affected region (13). In response, collateral venous channels may form to bypass the obstruction and restore outflow to more distal, patent venous structures (14). However, this compensatory collateralization is seldom sufficient, and venous hypertension persists. The result is progressive venous engorgement, which promotes interstitial edema in the adjacent brain parenchyma. Over time, this venous congestion contributes to neuronal dysfunction and structural changes, particularly in the white matter (12).

Cerebrovascular venous hypertension can impair venous drainage, elevate intracranial pressure, and reduce perfusion, leading to release of pro-inflammatory cytokines (12, 13, 15). This neuroinflammatory environment that may affect auditory processing centers, particularly when adjacent to congested venous structures like the sigmoid or transverse sinuses. The resulting inflammation and altered vascular-neural coupling can increase synaptic activity and lead to hyperexcitability of the auditory cortex (8). Over time, chronic hypoperfusion may contribute to axonal demyelination and impaired neural transmission (11), while persistent neuroinflammation and disrupted excitatory-inhibitory balance may sustain tinnitus symptoms (4, 6, 8). Therefore, venous hypertension may serve as both a mechanical and inflammatory trigger for non-pulsatile tinnitus.

We present two cases involving individuals who developed non-pulsatile tinnitus after COVID-19 vaccine but with normal audiometry. MR venography in both cases showed severe transverse sinus stenosis with loss of vascular flow in the sigmoid sinus. Hypoperfusion was implicated in tinnitus, consistent with neuroinflammation secondary to altered blood flow; the potential role of altered cochlear perfusion and anti-neuroinflammatory regimens are considered.

By presenting these two cases, we aim to encourage brainstorming and gather readers’ opinions—not only regarding the potential role of sigmoid sinus stenosis as a possible cause of tinnitus, but also on the broader impact that altered venous flow may have on cerebral circulation and its potential contribution to neuroinflammation.

Although the role of microvascular disease on the brain inflammation is well recognized (15), current research primarily focuses on arterial involvement. Based on the observations described, we hypothesize that venous stasis may play a more significant role than currently acknowledged.

We invite readers to share their perspectives on this hypothesis, which may help us determine whether adjustments to the clinical trial design are warranted.

## Materials and methods

This study included patients referred to the Neuromotor physiology laboratory at Santa Lucia Hospital for the persistent postural-perceptual dizziness and neuroinflammation clinical trial, but who failed to meet inclusion criteria because they were suffering from tinnitus without associated equilibrium disorders (human studies ethics approval # 0013698). We therefore offered the patients an opportunity to be enrolled in our clinical trial, already registered on Clinical Trial.gov with number NCT06718452, titled Tinnitus and Treatment with PEALUT.

The study was conducted in accordance with the Declaration of Helsinki. The patients signed a written consent to participate to the study and authorized the publication of their data in anonymized form for scientific purposes. The patients were screened following the clinical practice guideline for tinnitus by Tunkel et al. (16), and an MRI/MRV evaluation was performed to evaluate for underlying structural, neuroinflammatory, or vascular disorders (17), such as multiple sclerosis (6) or sigmoid sinus stenosis (10–12).

We used a questionnaire requesting that patients describe the characteristics of tinnitus (Table 1), followed by a 5 question online questionnaire (https://www.starkey.com/tinnitus/tinnitus-test). Moreover, if patients agreed, we also performed the Tinnitus Handicap Inventory (THI).

**Table 1:** The 5 questions to investigate tinnitus characteristics.

Question	Answer 1	Answer 2
Type of tinnitus	Pulsatile	Non-pulsatile
Duration	< 6 months	> 6 months
Intensity (Volume)	High	Low
Persistent	Yes all the day	No prevalently on evening/night
It affects everyday activity	No	Yes

### Audiological investigations

Pure Tone Auditory (PTA) test was performed in a silent cabin with earphones. Each ear was tested individually starting with the right side, without masking the contralateral side. Thresholds were tested at 125, 250, 500, 1,000, 2000, 4,000 and 8,000 Hz using the standard clinical ascending-descending procedure in 5 dB HL steps. Sensorineural Hearing Loss (SNHL) was classified following American Association Language -Hearing Association (ASHA) guidelines.

Tinnitus test was performed after assessing PTA. Each ear was tested sending a pure tone sound at 500, 1000, 2000, 4,000, 8,000 and 12,000 Hz with decibel defined based on the results of PTA; for example the sound was perceived at 35 dB in presence of mild SNHL.

Tympanometry and research of acoustic reflex. This test was performed to exclude damage in the middle ear that could be responsible of conductive or mixed hearing loss, i.e., otosclerosis or Meniere’s Disease. The testing was performed following ASHA guidelines (https://www.asha.org/policy/rp1988-00027/?srsltid=AfmBOooM5kpcQKK4btiJxfGLWIFo-rFFtnMHIplIsrnDai91_p5-vZQ6).

Speech perception test (SPT) was performed using earphones by asking patients to repeat the word that they have heard. Twenty Italian words extracted from a list of single words (mono- or bi-syllabic words of common use) that also contained minimal pairs. Words were individually presented in each ear without masking the contralateral ear, as previously described (18).

Auditory Brain Response (ABR) was recorded with an ICS CHARTR EP 200 (www.otometrics.natus.com) in clinical, automatic modality using a click stimulus with alternating polarity. The auditory perception level was set to the average hearing level at 2–4 kHz: <40 dB hearing level used a click stimulus at 70 dB; 40–60 dB hearing level used a click stimulus at 80 dB; and >60 dB hearing level used a click stimulus at 90 dB. Contralateral masking was applied if asymmetric responses were observed.

### Imaging

A 1.5 Tesla MRI with and without contrast was performed using standard protocols for brain investigations. If vascular anomalies were identified, venography was performed for better delineation of anatomic anomalies.

After these investigations, other conditions from which the tinnitus could arise (14) had been excluded. In the presence of idiopathic (primary) tinnitus (14) we prescribed umPEALUT Glialia 700 mg + 70, Epitech group, Saccolongo (PD). The product is an oral supplement cointaining co-ultramicronized palitoiletanolamide (PEA) and Luteolin (LUT) commonly used to treat neuroinflammatory conditions (ie Multiple Sclerosis).

### Treatment and posology

Patients were instructed on the correct method of taking umPEALUT. The supplement should be taken sublingually (under the tongue) and preferably with meals—ideally at breakfast and lunch. The treatment regimen consisted of two sachets per day for the first 30 consecutive days, followed by one sachet per day for the next 60 days.

Demographic info was collected.

## Results

### Case 1

A 59-year-old woman presented to the otolaryngology clinic suffering from bilateral non-pulsatile tinnitus that started 3 months earlier and persisted thereafter. The patient reported the onset of the symptoms 15 days after the second dose of COVID-19 vaccination. She denied cardiovascular disorders, diabetes or thyroid disease or any other major condition, including previous.

Audiologic evaluation demonstrated a bilateral mild high-frequency sensorineural hearing loss; specifically, audiometry showed thresholds in the right ear of 35 dB at 2000 Hz, 40 dB at 4000 Hz and 55 dB at 8000 Hz, and thresholds in the left ear of 35 dB at 1000 Hz, 30 dB at 2000 Hz, 50 dB at 4000 Hz and 45 dB at 8000 Hz. Tinnitus was identified at 10,000 Hz bilaterally. Speech perception test (SPT) scores were >90% at 45 dB. Auditory Brain Response (ABR) was bilateral unstructured with sensory layout (https://www.audiologyonline.com/articles/update-on-auditory-evoked-responses-17434).

**Figure 1:**
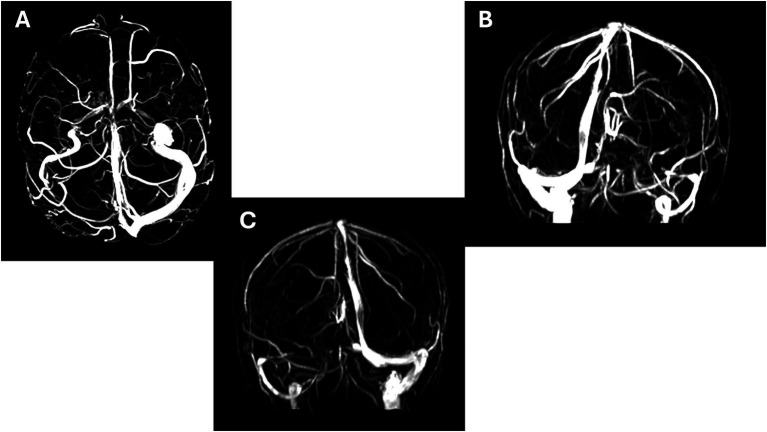
Magnetic resonance venography (MRV): Frontal **(A)** coronal from left and from right side **(B,C)** severe stenosis of sinus transversum, with stenosis of the sigmoid sinus and carotid on the same side.

The imaging findings showed significant vascular anomalies. An internal auditory canal and cerebellopontine angle protocol MRI with contrast was unremarkable, except for the presence of a small unspecific white matter hyperintensity in the right frontal lobe. However, magnetic resonance venography identified a right severe stenosis of the transverse sinus, with stenosis of the sigmoid sinus and ipsilateral carotid. The right-side vascular structures demonstrated compensatory dilatation (Figure 1).

Based on the hypothesis that hypoperfusion-related neuroinflammation might account for the onset of tinnitus, the woman was administered umPEALUT. The patient reported a subjective reduction of the intensity and frequency of the tinnitus after 3 months of therapy.

### Case 2

A 44-year-old woman presented to our clinic with a history of abrupt onset of right-sided, high pitched non-pulsatile tinnitus of 3 years duration that was unresponsive to behavioral interventions or home remedies. She was in excellent health status except for tinnitus.

The patient reported loud noise exposure at a nightclub the day before tinnitus onset. She also reported taking the third COVID-19 vaccine dose 1 week before tinnitus onset. The post nightclub non-pulsatile tinnitus persisted less than 24 h and then spontaneously remitted.

**Figure 2:**
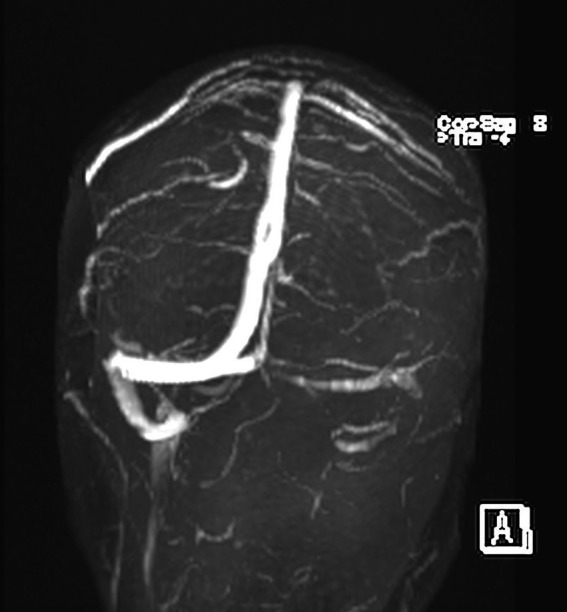
Magnetic resonance venography (MRV): the stenosis of the right sigmoid sinus in posterior cranium view (occipital).

Two days after the remission, the tinnitus recurred and persisted for days. Serial assessment of pure tone averages in 2021, 2022, 2023, and 2024 showed normal auditory thresholds. Speech perception test (SPT) scores were > 95% at 20 dB. ABR showed normal I, III and V waves bilaterally. Tinnitus was identified at 8,000 Hz in the right ear. Brain MRI was normal, but the MR venography identified stenosis of the right sigmoid sinus (Figure 2) without contralateral compensatory hyperplasia. The patient was treated using umPEALUT, and when reassessed at 90 days, she reported significant improvement of her tinnitus.

## Discussion

These two case reports highlight the utility of MRI venography in detecting vascular abnormalities linked to non-pulsatile tinnitus and suggest a potential approach for treating tinnitus when neuroinflammation, stemming from hypoperfusion, is suspected. Pereira et al. demonstrated that the use of a stent can improve the blood flow and consequentially brain perfusion (19) reducing pulsatile tinnitus; however, to the best of our knowledge there are no studies that analyzed/identified pharmacologic treatment for vascular sinus abnormalities in patients with non-pulsatile tinnitus. Although most literature on sigmoid sinus anomalies considers procedural interventions, the possibility of more conservative pharmacological approaches has intuitive appeal.

These preliminary results suggest a role for further investigation into the use of anti-neuroinflammatory agents such as PEA and Luteolin for treating idiopathic tinnitus (20, 21). A cannabinoid mimic molecule can reduce brain inflammation and reduce the activation of the auditory cortex (4, 6). Additionally, Luteolin might have improved the efficacy of PEA. Luteolin, a bioflavonoid extracted from fruits, has notable anti-inflammatory vessels proprieties (22); the reduction of vascular inflammation, which was sustained by the sigmoid sinus alteration (19, 23), might have improved the anti-neuroinflammatory effect of PEA.

### Mechanisms of tinnitus

Tinnitus arises from hyperactivation of the auditory cortex (4, 6). Several local (7, 8, 24) and systemic conditions (6, 24) can act as triggers. Neuroinflammation has been associated with tinnitus (25), and studies have linked tinnitus to COVID infection (26) or COVID-19 vaccination (27). In both instances, the underlying etiology is thought to involve micro-thrombosis (26) caused by the hypercoagulation induced by the spike protein (27) or related to the neuro-inflammatory processes that affect brain (28) because of pro-inflammatory effect of SARS-CoV2 spike protein (29). Both patients reported the onset of non-pulsatile tinnitus following COVID-19 vaccination; however, these patients also had history suggestive of acute or chronic peripheral auditory impairment. In Case 1, the patient had bilateral mild high-frequency sensorineural hearing loss, a known risk factor for non-pulsatile tinnitus. In Case 2, the patient experienced temporary non-pulsatile tinnitus after acoustic exposure at a nightclub, which resolved within 24 h. It therefore remains unclear whether these pre-existing factors were preconditions for the development of persistent tinnitus, or whether the vaccine alone could have triggered a hypercoagulable or neuroinflammatory response contributing to sigmoid sinus occlusion and symptom onset. Pro-inflammatory effects from vaccine-associated spike proteins might also contribute to auditory pathway changes in individuals with underlying peripheral auditory vulnerability. Given these potential contributing factors, the temporal link between COVID-19 vaccination and tinnitus onset must be interpreted with caution, and future investigations are needed.

Another important aspect of difference among the two case that must be underlined is that patient 1 had not only a sigmoid sinus stenosis but also a carotid stenosis. The presence of the two vascular occlusions might explain the bilaterality of tinnitus because the two stenotic vessels can trigger more neuroinflammation (venous–arterial blood deficit) and hyperactivate both auditory area.

### Tinnitus and neuro-inflammatory disorders

Tinnitus can also be a sign of neuro-inflammatory disorders like Multiple Sclerosis (6), related to demyelination in the auditory cortex or at the level of pons (30). This symptom can also be a harbinger of cardiovascular disease (31). For example, tinnitus in younger populations is associated with an increased risk of stroke (32). Carotid obstruction (internal artery) is one of the causes of tinnitus especially in patients over 65 (33). Increased flow turbulence into the carotid artery that indirectly affects the perfusion of the labyrinthine artery can cause cochlea hypoxia (34, 35). Depending on the severity and length of hypoxia, patients can develop hearing loss or tinnitus (36, 37).

**Figure 3:**
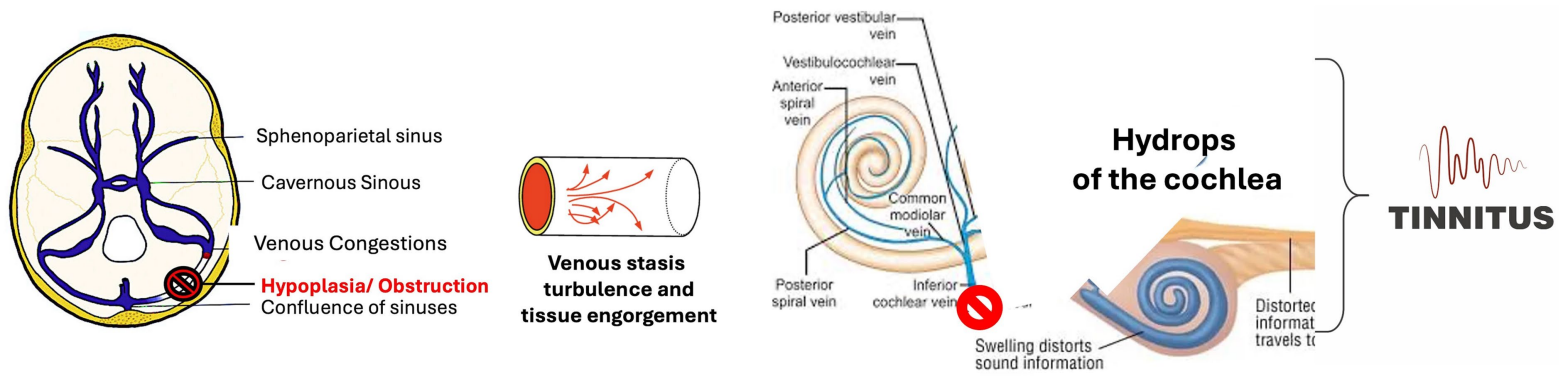
The drawing summarizes the peripheral origin of the tinnitus related to change of vascular flow because of sigmoid sinus hypoplasia/obstruction. The vestibular vein cannot correctly drain in the sigmoid sinus; this can cause the hydrops of the cochlea with the onset of tinnitus. Then because the sinus alteration induces neuroinflammatory processes, the tinnitus of peripheral origin might be sustained by a central cause.

Fluid dynamics are relevant to sigmoid sinus hypoplasia (17, 20, 37) as well as diverticula and other anomalies. The change in the venous drainage of the labyrinthine vein, which drains in sigmoid sinus, might induce hydrops into the cochlea with hair cells injury and death (35, 36) (Figure 3).

Thus, loss of inputs from the inner ear can contribute to hyperactivity in the auditory cortex through disinhibition. Furthermore, Li et al. showed that sigmoid sinuous hypoplasia induced changes in the white matter and brain inflammation (22); the presence of tinnitus might reflect inflammation of the temporal lobe rather than inner ear ischemia.

### Venous hypertension and neuroinflammation

In both cases, the patients experienced high-frequency tinnitus (10 kHz in Case 1 and 8 kHz in Case 2), suggesting a central origin of the symptom. Research indicates that high frequencies are tonotopically organized in the auditory cortex, with a distribution from anterior (low frequencies) to posterior (high frequencies) along the superior temporal gyrus, extending into the lateral sulcus and Heschl’s gyri. Since both patients perceived high-frequency sounds, this migh point to a particular vulnerability of the posterior ventral portion of the auditory cortex to neuroinflammation. Given that neuroinflammation can affect any region of the brain, the apparent predilection for involvement of the ventral posterior auditory cortex—compared to its dorsal anterior counterpart—would warrant further investigation. This area is drained by the deep middle cerebral vein, which connects to the straight sinus via the anterior cerebral vein. We hypothesize that this area’s proximity to the vein exit may increase its susceptibility to neuroinflammation.

Zhou et al. (38) speculated that sigmoid sinus hypoplasia or stenosis may contribute to an increase of the intracranial venous pressure. Long-term venous hypertension could reduce brain perfusion and disrupt the intracranial microvasculature (38). Hypoperfusion has an important role in the development of white matter hyperintensities, and hypoxia can cause altered mitochondrial metabolism and death of oligodendrocytes (39). Mitochondrial dysmetabolism induce an excess of reactive oxygen species (ROS), inducing Disease Associated Microglia (DAM) (40), formerly name M1 -pro inflammatory microglia, which are responsible of brain neuroinflammation (41).

Hypoperfusion of the sigmoid sinus, or Transverse Sigmoid Sinus syndrome, leads to increased resistance to venous outflow, which elevates venous hypertension and intracranial pressure. This condition induces mechanical stress on vascular walls and triggers endothelial dysfunction. As a result, inflammatory pathways become activated, including release of cytokines such as IL-6, TNF-*α*, and vascular endothelial growth factor (VEGF). Concurrently, impaired venous outflow can cause localized ischemia or hypoxia in brain tissues, activating inflammatory signaling pathways associated with hypoxia-inducible factors, leading to microglial activation. Additionally, TSS may impair the glymphatic system, essential for removing brain waste, leading to an accumulation of neurotoxic metabolites such as amyloid-*β* and tau proteins. This accumulation can exacerbate neuroinflammatory conditions and contribute to the onset of symptoms like tinnitus, cognitive dysfunction, and headaches.

It is important to emphasize that stenosis and hypoplasia of the sigmoid sinus are distinct conditions, and the presence of symptoms can help differentiate between them (42). Wang et al. recently demonstrated that in cases of venous hypoplasia—a congenital condition—collateral vessels typically appear normal and provide adequate perfusion to the area. In contrast, in cases of stenosis, both the collateral vessels and their flow are abnormal, and patients experience symptoms. This suggests that congenital conditions are generally well compensated, whereas acquired conditions lead to vascular alterations and clinical symptoms. The work of Wang et al. has provided valuable clarification of these important findings.

**Figure 4:**
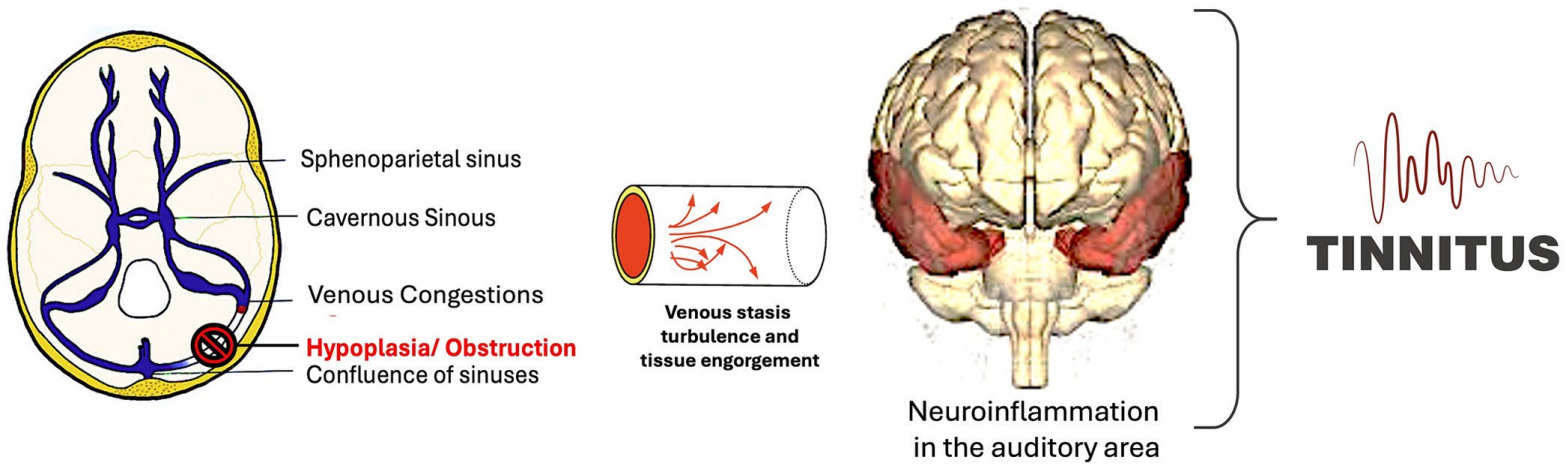
Schematic demonstrating the central origin of the tinnitus related to change of vascular flow because of sigmoid sinus hypoplasia/obstruction. Brain neuroinflammation from a vascular origin, might, in predisposed patients, induce hyperactivation of the temporal lobe with tinnitus onset.

Given the labyrinthine vein discharge in the sigmoid sinus, the stenosis of the latter could cause tinnitus through both inner ear damage (Figure 3) and brain neuroinflammation that sustains the hyperactivation of auditory area (Figure 4).

These mechanisms remain speculative, but the resolution of the symptom after the use of palmitoylethanolamide (PEA) is of interest. This molecule modulates the microglia state versus their anti-inflammatory phenotype (M2 microglia) reducing brain neuroinflammation (20, 21). PEA has been widely used to treat brain neuroinflammation (20, 21) so the rationale is based on anti-neuroinflammatory effect that might alleviate tinnitus although controlled studies are needed (10). Additionally, correcting venous stenosis with stenting tackles may address root cause of neuroinflammatory activation, while therapies such as antioxidants and VEGF inhibitors, may mitigate inflammatory triggers.

### Limitations of the study

Larger samples are needed to confirm the role of sigmoid sinus stenosis in tinnitus onset and randomized trials would be ideal for unbiased assessment of palmitoylethanolamide as a neuro-inflammation modulator. Furthermore, we did not have baseline imaging data to identify the status of the transverse and sigmoid sinus before the tinnitus onset. In both cases the patients did not fill in a tinnitus questionnaire but rather described the discomfort related to the symptom. The link between sigmoid sinus hypoplasia/stenosis and tinnitus is consistent with previous studies (12, 19, 23), but further investigations are needed. Such work should involve systemic anti-inflammatory agents such as NSAIDs and glucocorticoids, alongside experimental therapies like palmitoylethanolamide (PEA) and luteolin, which can potentially help modulate inflammation caused by venous hypertension. Another important limitation is the lack of Tinnitus Handicap Inventory (THI) questionnaire assessment; absence of this quantitative data with a validated instrument makes the findings regarding tinnitus resolution more difficult to interpret, based on the potentially fluctuating character of tinnitus perception. Further studies including the THI assessment are needed. Moreover, audiological evaluations were performed only at baseline; however, to better assess the treatment’s benefits, they should also be conducted at the end of the treatment. Finally, we did not test coagulation in these two patients even because vaccine was done several time before we observed them; considering that hypercoaugulation post vaccine is a temporary condition, the coagulation test should be immediately performed at symptom onset to identify it.

## Conclusion

Sigmoid sinus vascular abnormalities are a potentially underrecognized cause of tinnitus. The alteration of venous flow might affect the inner ear causing hydrops or reduce scavenging of ROS, resulting hair cell injury and tinnitus. Alternatively, hypoperfusion caused by the altered vessel anatomy might induce neuro-inflammation, explaining auditory hyperactivation with tinnitus (10). Yet another possibility is a combination of these causes.

It is important to underline that we discussed a hypothesis on the base of preliminary observations.

The findings of this brief report, though preliminary, provide a rationale for further clinical investigation assessing the efficacy of anti-neuroinflammatory therapy for tinnitus, capturing relevant data on anatomy, audiometry, and validated patient-reported outcomes.

## Data Availability

The raw data supporting the conclusions of this article will be made available by the authors, without undue reservation.
