# Supplementary material for: Anti-neuroinflammatory therapy for non-pulsatile tinnitus in patients with sinus vascular anomalies: preliminary result on two cases
Source: Front Neurol. 2025 Jul 23;16:1558196. doi: 10.3389/fneur.2025.1558196 (PMC12325037; doi:10.3389/fneur.2025.1558196)
Supplement: Supplementary file 1 [file Table_1.docx]

| **Question** | **Answer 1** | **Answer 2** |  |
| --- | --- | --- | --- |
| *Type of tinnitus* | Pulsatile | Non-pulsatile | |
| *Duration* | < 6 months | > 6 months |  |
| *Intensity (Volume)* | High | Low |  |
| *Persistent* | yes all the day | no prevalently on evening/night | |
| *It affects everyday activity* | No | Yes |  |
